# Zone-specific hepatocytes orchestrate the early onset of host immune defenses during *Staphylococcus aureus* bloodstream infection

**DOI:** 10.3389/fimmu.2026.1776887

**Published:** 2026-04-30

**Authors:** Obiageli V. Nwofor, Alexander Leipold, Qian Chen, Robert Geffers, Antoine-Emmanuel Saliba, Oliver Goldmann, Eva Medina

**Affiliations:** 1 Infection Immunology Research Group, Helmholtz Centre for Infection Research (HZI), Braunschweig, Germany; 2 Helmholtz Institute for RNA-based Infection Research (HIRI), Helmholtz Centre for Infection Research (HZI), Würzburg, Germany; 3 Genome Analytics, Helmholtz Centre for Infection Research (HZI), Braunschweig, Germany

**Keywords:** acute-phase response, bloodstream infections, BMPER, hepatocytes, Kupffer cells, liver zonation, *Staphylococcus aureus*

## Abstract

**Background:**

Bloodstream infections (BSI) are life-threatening conditions initiated by pathogens entering the circulation, with *Staphylococcus aureus* among the most lethal causative agents. The liver is the primary site for the initial detection and clearance of blood-borne pathogens. Emerging evidence indicates that the hepatic immune response to pathological insults is influenced by liver zonation, exhibiting both spatial and cellular specialization. Because direct experimental evidence linking liver zonation to the immune response to BSI is currently lacking, this study aimed to characterize the compartmentalization of the early immune response in the liver to blood-borne *S. aureus*.

**Methods and results:**

Using an intravenous infection model, we found that the liver captured ~90% of circulating *S. aureus* within 4 h and significantly reduced bacterial loads by 24 h, indicating rapid activation of intrahepatic immune defenses. Bulk RNA sequencing revealed strong induction of acute-phase and interferon-associated genes at 4h of infection, alongside elevated levels of IL-6, IL-1α/β, TNF-α, and IFN-γ. Single-cell transcriptomics identified hepatocytes as the principal responders, displaying pronounced zone-specific transcriptional programs along the porto-central axis. Periportal and midzonal hepatocytes showed the highest activation, whereas pericentral hepatocytes exhibited comparatively reduced transcriptional responses. Among the most highly upregulated genes was *Bmper*, encoding a regulator of bone morphogenetic proteins (BMPs) signaling that has not previously been linked to bacterial infections. *Bmper* induction was selective for periportal and midzonal hepatocytes. Chemokine production was likewise compartmentalized, with hepatocytes as the source of the neutrophil chemoattractant CXCL1, whereas Kupffer cells and liver sinusoidal endothelial cells expressed monocyte-attracting CCL chemokines. An expansion of Kupffer cells was also observed in infected livers, likely driven at least in part by the local proliferation of resident Kupffer cells.

**Conclusions:**

This study demonstrates that the liver immune response to *S. aureus* BSI is highly compartmentalised, with hepatocytes acting as central orchestrators of the initial immune defence and exhibiting zone-specific transcriptional patterns. The strong, selective induction of *Bmper* suggests a previously unrecognized link between BMPs signaling and the acute-phase response to infection. Further investigation into the role of BMPER in the acute-phase response to BSI may reveal new strategies to enhance hepatic immunity or mitigate inflammation-induced liver injury.

## Introduction

Bloodstream infections (BSI) are life-threatening conditions caused by the presence of pathogenic microorganisms in the circulation, and they remain a major cause of morbidity and mortality worldwide (1). The increasing prevalence of antimicrobial resistance, the widespread use of immunosuppressive therapies, and the demographic shift towards an ageing population are expected to further increase the population at-risk for these infections (2). This trend underscores the urgent need for innovative preventive and therapeutic strategies. A detailed understanding of the mechanisms underlying each stage of BSI pathogenesis is crucial for the rational design of effective, targeted interventions.

The pathogenesis of BSI is multifaceted and typically involves the systemic dissemination of pathogens from a primary site of infection through the bloodstream (3). Upon entering the circulation, pathogens are rapidly filtered and neutralized by immune defense mechanisms within the liver (4–7). The hepatic microarchitecture, comprising sinusoidal endothelial cells, parenchymal cells, and resident macrophages (Kupffer cells), enables the efficient clearance of circulating pathogens (8). Compromised liver function significantly impairs this defensive capacity, leading to persistent bacteraemia and poor clinical outcomes (9). In addition to its role in pathogen clearance, the liver is the principal organ responsible for producing acute-phase proteins, which are critical components of the immune response during infection and inflammation (10–13). Acute-phase proteins are primarily synthesized by hepatocytes and orchestrate systemic inflammation and coagulation, promote pathogen clearance, modulate immune cell migration, and facilitate tissue repair, thereby reinforcing the liver as a central hub of systemic immune regulation (10–13).

Emerging evidence indicates that the liver’s response to pathological insults is highly compartmentalized, involving both spatial and cellular specialization (14, 15). The liver is structurally organized into zones along its lobules, each characterized by distinct metabolic, immune, and detoxification functions (16, 17). Specifically, the hepatic lobule is divided into periportal, midzonal, and pericentral regions (14, 15). Liver zonation may play an important role in shaping the immune response to BSI. For example, the periportal region is the first site to be exposed to incoming blood-borne pathogens. This zone is particularly enriched with Kupffer cells, which are key frontline phagocytes that intercept blood-borne pathogens arriving via the portal vein (18, 19). This strategic location enables the rapid detection and clearance of microbes upon entry the liver before they can spread deeper (18, 19). Furthermore, periportal, midzonal, and pericentral hepatocytes differ metabolically and transcriptionally, which may influence their response to infection (14, 15, 17, 20). The interplay among these regions may dictate the efficacy of pathogen clearance, the magnitude of inflammatory responses, and whether the infection resolves or escalates to systemic dissemination. A deeper mechanistic understanding of the role of liver zonation in the response to BSI could guide the development of spatially targeted therapeutic strategies that exploit zonal specialization to improve treatment efficacy.

*S. aureus* is one of the most common pathogens responsible for BSI (21–25). *S. aureus* can enter the bloodstream either through a primary infection focus, such as a skin wound, or through direct inoculation via an infected indwelling device (21). The earliest immune response to blood-borne *S. aureus* takes place in the liver (21). Upon entering the circulation, Kupffer cells rapidly capture and phagocytose circulating *S. aureus*, often before other systemic organs mount a response (26–28). This immediate clearance is essential for controlling the bacterial burden, as delayed removal can lead to uncontrolled systemic infection (21). *S. aureus* BSI also triggers a robust acute-phase response, characterized by increased production of acute-phase proteins (29). The central importance of the liver in *S. aureus* BSI is underscored by clinical observations showing that patients with chronic liver disease are more susceptible to *S. aureus* and experience higher mortality following infection (30). This indicates that hepatic dysfunction significantly influences infection outcomes (30).

Although direct experimental evidence linking liver zonation to the immune response against *S. aureus* BSI is currently lacking, the well-established influence of liver zonation on immune cell specialization and function suggests that it may be a key determinant in modulating the host response and shaping the progression of infection. Against this backdrop, the present study aimed to define the compartmentalization of the early immune response in the liver to blood-borne *S. aureus.* By integrating bulk and single-cell transcriptomic profiling, cytokine analyses, and flow cytometry, we have identified zonal hepatocyte populations, particularly periportal and midzonal hepatocytes, as pivotal coordinators of the initial immune response to *S. aureus* BSI. Furthermore, this study revealed the bone morphogenic protein-binding endothelial regulator (BMPER) as a potential novel mediator of the acute-phase response during BSI.

## Materials and methods

### Bacterial strains and growth conditions

The *S. aureus* strain SH1000 was used in this study (31). The bacteria were cultured in Brain Heart Infusion (BHI) medium (Roth) at 37 °C with continuous shaking at 120 rpm until the mid-log growth phase was reached. The bacteria were then harvested by centrifugation, washed with sterile PBS, and diluted to the required concentration.

### Animals

Pathogen-free female C57BL/6 mice were purchased from Charles River (Germany). The mice were housed under specific pathogen-free (SPF) conditions in the animal facility of the Helmholtz Centre for Infection Research. The animals were kept in individually ventilated cages with autoclaved bedding and nesting material.

### Infection model

Mice were inoculated with approximately 5 x 10^7^ CFU of *S. aureus* SH1000 in 100 µl of PBS via a lateral tail vein and euthanized by CO_2_ asphyxiation at 4 h or 24 h after bacterial inoculation. To determine the bacterial burden in liver, kidneys, and lungs, these organs were removed and homogenized in PBS. The numbers of CFU were determined by plating 10-fold serial dilutions on blood agar plates and incubating them for 24 h at 37 °C.

### RNA extraction and library preparation for bulk RNA sequencing

Total RNA was extracted from liver tissue of 5 mice from independent experiments using TRIZOL (Invitrogen), following mechanical disruption with a Teflon pestle. Phase separation was achieved by the addition of 200 µl of chloroform, followed by centrifugation for 10 min at 12,000 × *g* at 4 °C. The upper phase containing the RNA was transferred to a new tube, after which the RNA was precipitated by addition of 500 µl of isopropyl alcohol, followed by centrifugation for 10 min at 12,000 × *g* at 4 °C. After removing the supernatant, the RNA pellet was washed with 1 ml of 75% ethanol, centrifuged for 10 min at 12,000 × *g* at 4 °C and then air-dried for 5 min. The pellet was then dissolved in 50 µl RNase-free water, and residual DNA was removed using DNase treatment with TURBO™ DNase (Thermo Fisher Scientific). RNA quality was assessed using a NanoDrop spectrophotometer (Thermo Fisher Scientific) and an Agilent 2100 Bioanalyzer (Agilent Technologies).

RNA-seq libraries were generated using the Dynabeads^®^ mRNA DIRECT™ Micro Purification Kit (Thermo Fisher Scientific) for mRNA isolation, followed by the *NEBNext*^®^*Ultra*^™^*II Directional RNA* Library Prep Kit for Illumina (New England Biolabs), according to the manufacturer’s protocol. The libraries were then sequenced on a NovaSeq 6000 (Illumina) using the NovaSeq 6000 SP Reagent Kit (100 cycles), generating an average of 30 million reads per sample.

### Bioinformatic analysis of RNA sequencing data

The raw sequencing reads were quality filtered and trimmed for Illumina adapter contamination using fastq-mcf with a minimum quality threshold of 20. The filtered reads were then mapped to the *Mus musculus* reference genome (assembly GRCm38.p6, GCA_000001635.26) using STAR (Spliced Transcripts Alignment to a Reference). The resulting alignments were output in BAM format, and sorted by genomic coordinates. Subsequently, read counts were generated using featureCounts.

### Statistical analysis of transcriptional data

Differentially expressed genes (DEGs) were identified using the DESeq2 package in R with the threshold set to *p-*value < 0.05 and log_2_ fold change > 2 or < -2. Lists of all DEGs between conditions were used as input for a gene ontology (GO) enrichment analysis to identify association of gene sets with specific biological pathways using DAVID (32, 33) and SRplot (34). Volcano plots showing DEGs between conditions were generated using GraphPad Prism version 10.2.3. Principal component analysis (PCA) and heatmap visualization were performed using the MetaboAnalyst platform (35).

### Cytokines and chemokines quantification

Cytokine levels in liver homogenates were measured using the LEGENDplex™ Mouse Inflammation Panel (13-plex) (BioLegend), according to manufacturer’s instructions. CXCL1 levels were quantified in liver homogenates by ELISA (Abcam), following the manufacturer’s protocol.

### Isolation of liver cells

The mice were euthanized by CO_2_ asphyxiation and their livers were perfused with ice-cold PBS by inserting a cannula into the portal vein. After perfusion, the liver was removed, cut into small pieces, and incubated in a digestion solution containing 10% FCS, DNase1 (100 µg/ml) and collagenase IV (1 mg/ml) in a thermostatic bath at 37 °C for 45 min with stirring at 100 rpm. The enzymatic reaction was then stopped by adding medium containing 10% FCS, after which the liver was passed through a 70 µm filter. The digested tissue was pelleted by centrifugation at 300 x *g* for 5 min. Red blood cells were lysed using lysis buffer at room temperature for 5 min. The resulting cell pellets were resuspended in 33% Percoll, gently overlaid with PBS, and centrifuged at 300 x *g* for 20 min. The cells were collected from the bottom layer and used for further assays.

### Single-cell RNA sequencing

Liver cell suspensions prepared from five uninfected mice, five *S. aureus-*infected mice at 4 h of infection, and five mice at 24 h of infection were pooled and loaded onto the 10x Chromium Single Cell instrument (10x Genomics). Libraries were prepared from the single-cell suspensions using 3’scGEM-X v4 1-plex 20k, according to the manufacturer’s protocol (10x Genomics). Sequencing was performed on an Illumina NovaSeq X 1,5B 300 cycle sequencer (Illumina), with a sequencing depth of 200 million reads per sample.

### Single-cell RNA sequencing data analysis

Raw sequencing data were demultiplexed and quality checked using the CellRanger ‘mkfastq’ script (10x Genomics, v8.0.1). Gene-barcode matrices were generated by alignment against the mm10 Reference genome (GENCODE vM23/Ensembl98) and transcript quantification using the CellRanger ‘count’ script. Downstream analysis was performed in R (v4.2.3) using the Seurat R package (v5.1.0). Seurat objects of the three conditions were merged, and then count data was normalized to library-size (scale.factor = 10000) and log-transformed. For initial quality control, barcodes with a mitochondrial count fraction of more than 0.25 were removed. Highly variable features were selected based on variance stabilizing transformation, scaled, and subjected to principal component analysis (PCA). To account for batch effects between the conditions, the Seurat reciprocal principal component analysis (RPCA) workflow was used. Uniform manifold approximation and projection (UMAP) was used to compute a two-dimensional embedding based on the first ten RPCA components. Unsupervised Louvain clustering based on a nearest-neighbour graph was applied with a resolution of 0.5. Clusters exhibiting high mitochondrial count fraction, low UMI counts, and a low number of expressed genes were removed. Dimensional reduction, batch correction, and clustering was repeated as described, with a clustering resolution of 0.8. Genes marking clusters were computed using the FindAllMarkers implementation of the Wilcoxon rank-sum test in Seurat. Clusters were annotated based on expression of known marker genes. Differentially expressed genes between conditions were identified using FindMarkers (min.pct = 0.25). Genes were deemed differentially expressed with an adjusted *p*-value < 0.05 and log2 fold change > 2. Kupffer cells were subset and dimensional reduction, batch correction was applied analogously. Cell cycle scores were calculated and cell cycle phase (G_1_, G_2_/M, S) was assigned for each cell based on gene sets from Tirosh et al. (36) using the CellCycleScoring function.

### Data and code availability

The raw and processed scRNA-seq data generated in this study have been deposited in the NCBI Gene Expression Omnibus (GEO) under accession number GSE312076 (https://www.ncbi.nlm.nih.gov/geo/query/acc.cgi?acc=GSE312076). The bulk RNA-seq data have been deposited in Harvard Dataverse (https://doi.org/10.7910/DVN/34G5OW).

### Quantitative RT-PCR

Real-time RT-PCR assays were performed using the SensiFAST SYBR No-ROX one-step kit (BioCat). The PCR reactions were carried out using the following thermal cycling conditions: reverse transcription at 45 °C for 15 min, polymerase activation at 95 °C for 15 min, followed by 40 cycles of denaturation at 95 °C for 20 s, annealing at primer-specific temperatures for 20 s, and extension at 72 °C for 20 s. The primer sequences for *Bmper* gene were as follows: forward, CCTGCTGTGAACGATGCAAAGG; reverse, ACCTCAGACTCTGTCACCACAC. The gene encoding *β*-actin (*Actb)* served as reference gene. The primer sequences for *Actb* gene were as follows: forward, TGG AAT CCT GTG GCA TCC ATG AAA C; reverse, TAA AAC GCA GCT CAG TAA CAG TCC G. Relative gene expression levels were calculated using the comparative threshold cycle (ΔΔCT) method as described by Pfaffl (37).

### Flow cytometry analysis

Cells were incubated with anti-mouse CD16/32 (Miltenyi Biotec) for 10 min at RT to block Fc receptors and subsequently stained with antibodies against surface antigens for 30 min at 4 °C. After staining, cells were washed with PBS containing 10% FCS and analyzed on an LSRII cytometer (Becton Dickinson). The following antibodies have been used in this study: anti-mouse CD11b-PE/cyanine (BioLegend), anti-mouse Ly6C-Brilliant violet 421 (BioLegend), anti-mouse Ly6G- Brilliant violet 711 (BioLegend), and anti-mouse Tim4 Alexa fluor 647. Gatting strategies are shown in **Supplementary Figure S1**.

### Statistical analysis

Results are expressed as mean ± SD of biological replicates. Data analyses were performed using GraphPad Prism Version 10.6.1. For comparison between two groups, two-tailed t test was used. For multiple-group comparisons, one-way analysis of variance (ANOVA), followed by Tukey’s *post hoc* test, was applied. A *p*-value < 0.05 was considered statistically significant. Flow cytometry data were analysed using FlowJo 10.10.0 software.

## Results

### Blood-borne *S. aureus* induces acute-phase and interferon-associated gene expression in the liver

**Figure 1:**
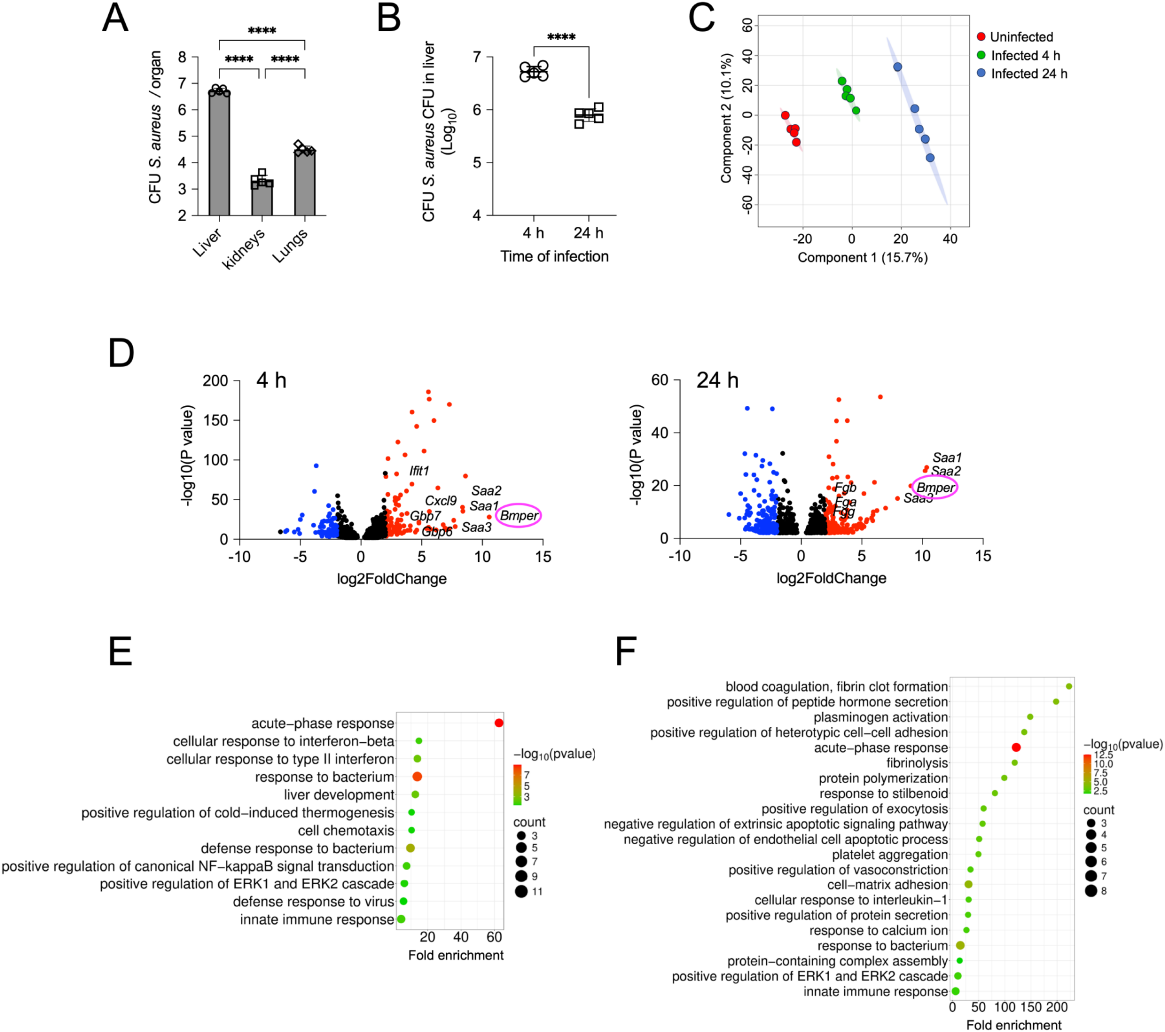
*S. aureus* BSI induces early transcriptional activation of acute-phase and interferon responses in the liver. **(A)** Quantification of bacterial burden in systemic organs at 4 h after intravenous inoculation with *S. aureus*. Each bar represents the mean ± SD. One representative experiment out of three is shown. ****p < 0.0001. **(B)** Quantification of bacterial loads in the liver at 4 h and 24 h after intravenous inoculation with *S. aureus*. Each symbol represents an individual mouse; horizontal lines indicate mean values ± SD. One representative experiment out of three is shown. ****p < 0.0001. **(C)** PCA of global gene expression in liver samples from uninfected mice and mice infected with *S. aureus* for 4 h or 24 h. Each point represents an individual liver sample, coloured according to infection status. Percentages on the axes indicate the proportion of total variance explained by each principal component. **(D)** Volcano plots of DEGs in the livers of infected mice at 4 h (left panel) and 24 h (right panel) of infection, compared with uninfected controls. The x-axis shows the log_2_ fold change in expression (infected vs. control), and the y-axis represents the –log_10_ of the adjusted *p*-value. Each point corresponds to a single gene. Significantly upregulated genes in infected samples are shown in red, downregulated genes in blue, and non-significantly regulated genes are indicated in black. **(E, F)** Bubble plots showing the top enriched GO terms among DEGs identified by RNA-seq in the livers of *S. aureus*-infected mice at 4 h **(E)** and 24 h **(F)** of infection, compared with uninfected samples. The y-axis lists enriched GO terms, and the x-axis shows the enrichment score (-log_10_ of the adjusted *p*-value). Bubble size reflects the number of genes associated with each GO term, while colour intensity indicates the significance level of enrichment.

An intravenous murine infection model was used to investigate the liver’s response to blood-borne *S. aureus.* Consistent with previous studies (26, 27), the liver sequestered the vast majority (approximately 90%) of the inoculated bacteria at 4 h of infection, whereas only minimal bacterial burdens were detected in other systemic organs, such as the kidneys and lungs (**Figure 1A**). Despite this substantial bacterial burden, the liver markedly reduced bacterial loads by 24 h of infection (**Figure 1B**), indicating the rapid and efficient activation of local immune defences.

To gain deeper insight into the liver’s early response to blood-borne *S. aureus*, we performed transcriptomic profiling of liver from infected mice at 4 h and 24 h after bacterial inoculation using RNA-seq. A heatmap of gene expression levels revealed distinct transcriptional patterns in the livers of infected mice, compared to uninfected controls (**Supplementary Figure S2**). These differences were further supported by principal component analysis (PCA), which demonstrated clear separation between the three groups (uninfected, infected for 4h, and infected for 24 h) along the first principal component (PC1) (**Figure 1C**). DEGs between infected liver samples and uninfected controls were identified using DESeq2 analysis. Genes were classified as upregulated if they exhibited a log_2_ fold change > 2 with and adjusted *p*-value of < 0.05, and as downregulated if they showed a log_2_ fold change < −2 and adjusted *p*-value of < 0.05. A total of 204 genes (125 upregulated and 79 downregulated) displayed significant changes in expression at 4 h of infection (**Supplementary Table S1**), and a total of 146 genes (71 upregulated and 75 downregulated) at 24 h of infection (**Supplementary Table S2**), relative to uninfected controls. These gene expression data are shown in the volcano plots in **Figure 1D**. Among the most highly upregulated genes at 4 h of infection were those encoding mediators of the acute-phase response, including serum amyloid A1 (*Saa1*), A2 (*Saa2*), and A3 (*Saa3*), interferon-induced genes such as *Cxcl9* and *Ifit1*, and the neutrophil chemoattractant *Cxcl1* (**Figure 1D** and **Supplementary Table S1**). At 24 h of infection, the most highly expressed genes were predominantly those encoding acute-phase proteins and mediators of the haemostatic response (**Figure 1D** and **Supplementary Table S2**). Notably, *Bmper*, which encodes the bone morphogenic protein-binding endothelial regulator BMPER, was one of the most strongly upregulated genes in the infected liver at both time points (**Figure 1D**). BMPER is an important regulator of bone morphogenic proteins (BMPs) signaling and is widely recognized for its role in vascular biology and endothelial cell function (38–40). However, its specific involvement in the acute-phase response of the liver during bacterial BSI has not been previously reported.

Gene ontology (GO) enrichment analysis was conducted to characterize the biological processes in the liver that are influenced by *S. aureus* BSI. The most significantly enriched GO term at 4 h of infection was the acute-phase response, followed by cellular responses to interferons (**Figure 1E**). By 24 h of infection, in addition to the acute-phase response, pathways related to coagulation, plasminogen activation, and fibrinolysis were also significantly enriched (**Figure 1F**). This indicates a progressive engagement of haemostatic networks in response to infection.

**Figure 2:**
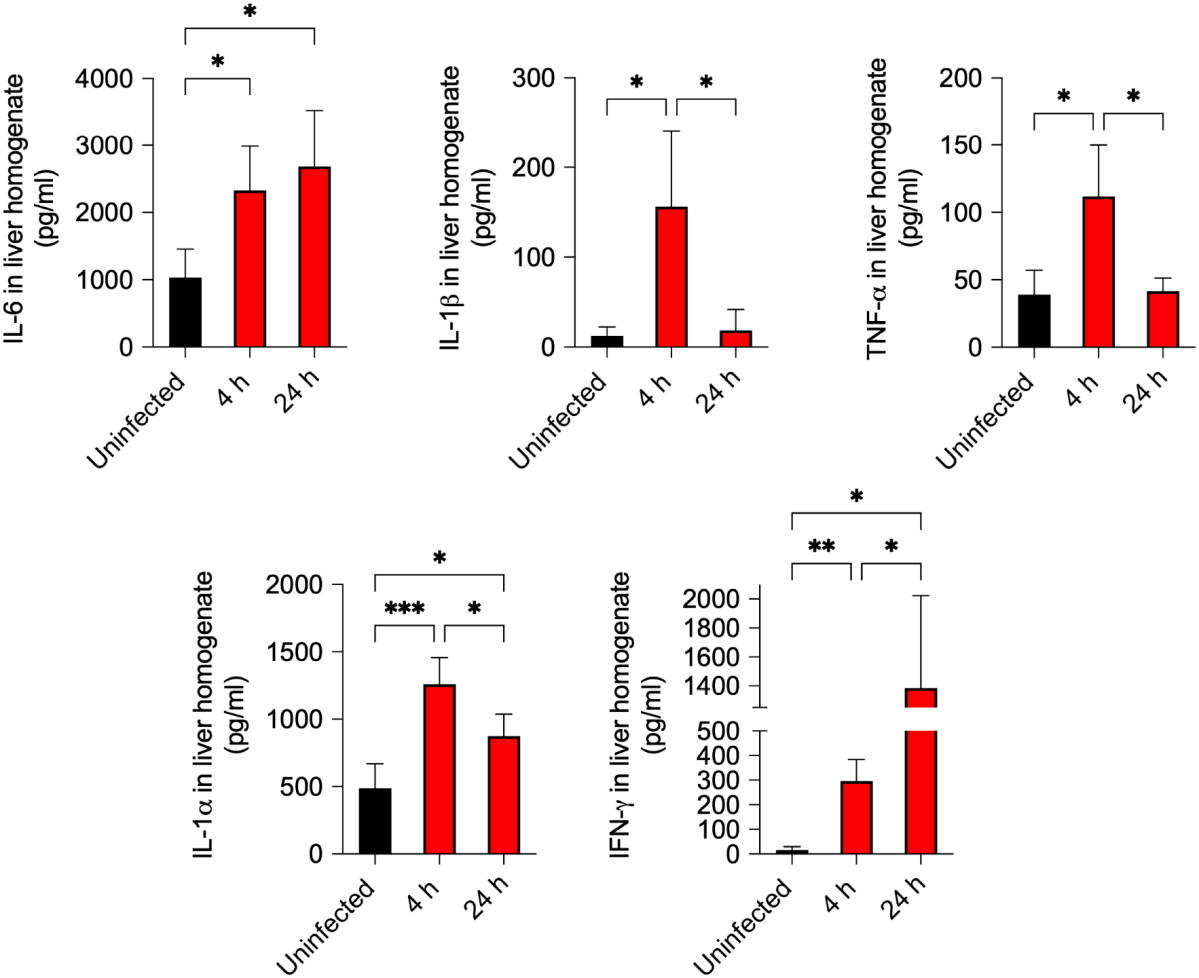
Increased levels of pro-inflammatory cytokines in the liver of *S. aureus*-infected mice. Livers were collected from uninfected mice and from mice at 4 h and 24 h after intravenous inoculation with *S. aureus*. Cytokine concentrations, including IL-6, IL-1β, TNF-α, IL-1α, and IFN-γ, were quantified in liver homogenates by flow cytometry. Data are presented as mean ± SD from n = 5 biological replicates. *p < 0.05, **p < 0.01, ***p < 0.001.

Hepatocytes are the principal producers of acute-phase proteins (41), whose synthesis is stimulated by pro-inflammatory cytokines such as IL-6, IL-1β and TNF-α (11, 42). These cytokines are mainly secreted by Kupffer cells and possibly by endothelial cells during infection. The early induction of genes encoding acute-phase proteins in the liver, noticeable as soon as 4 h after *S. aureus* inoculation, suggests that the cytokines driving this response are produced locally during the early phase of infection. Significantly increased concentrations of IL-6, IL-1β, IL-1α, and TNF-α were indeed detected in the liver at 4 h of infection compared with uninfected controls (**Figure 2**). The kinetics varied among the cytokines. Specifically, IL-6 and IL-1α levels remained elevated at 24 h of infection, whereas the levels of IL-1β and TNF-α levels returned to the baseline. In addition to these pro-inflammatory cytokines, IFN-γ levels were elevated in liver homogenates at both 4 and 24 h of infection (**Figure 2**).

### scRNA-seq identifies hepatocytes as the predominant liver cell type responding to blood-borne *S. aureus*

**Figure 3:**
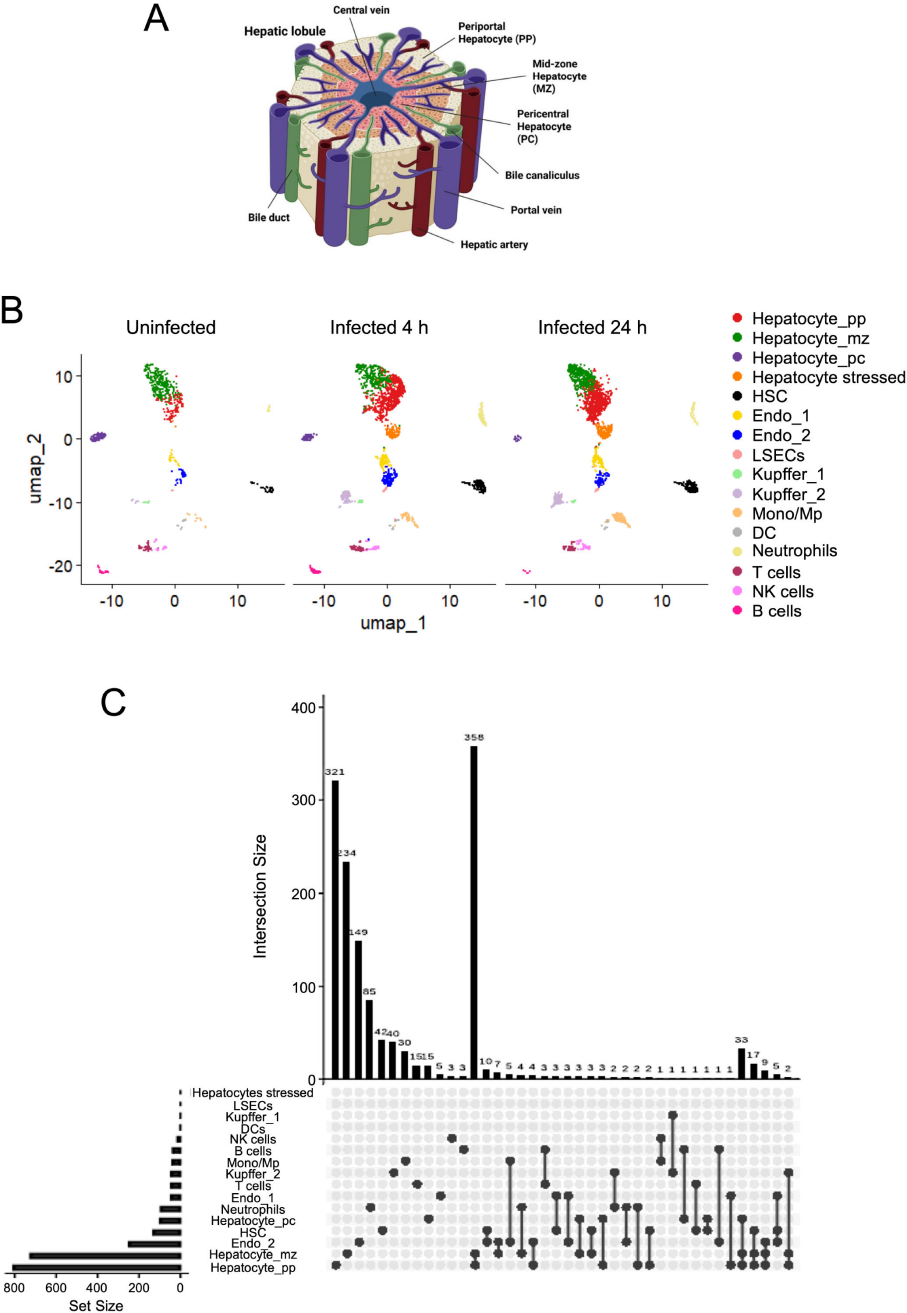
Hepatocytes represent the principal liver cell type responding to blood-borne *S. aureus*. **(A)** Schematic illustration of the spatial zonation of hepatocytes along the porto-central axis of the liver lobule. The periportal zone (pp) is located near the portal triad, the midzonal region (mz) represents a transition area, and the pericentral zone (pc) is adjacent to the central vein. Created with BioRender.com. **(B)** UMAP plot showing transcriptionally distinct cell clusters identified by scRNA-seq of liver samples from uninfected mice and mice intravenously infected with *S. aureus* (4 h and 24 h of infection). Each point represents a single cell. Cells are coloured based on cluster identity as determined by unsupervised clustering. **(C)** UpSet plot of DEGs across liver cell clusters in response to *S. aureus* infection at 4 h after intravenous inoculation. The bar chart on the left indicates the total number of DEGs per cell cluster, while the dot-matrix shows overlapping DEGs combinations across cell clusters. Rows with filled dots under two or more columns denote DEGs shared among multiple clusters, and connected filled dots indicate common DEGs among the corresponding clusters. The bar chart at the top represents the size of each intersection.

The liver comprises several cell types, including hepatocytes, hepatic stellate cells (HSC), Kupffer cells, and liver sinusoidal endothelial cells (LSECs), which work together to maintain metabolic homeostasis, detoxification, and immune surveillance (43). Parenchymal cells are primarily hepatocytes, accounting for approximately 70-80% of the liver mass, and performing many of its essential metabolic and biosynthetic functions (44). Kupffer cells are the liver’s resident macrophages and represent the largest population of tissue-resident macrophages in the body. They recognise pathogenic stimuli entering through the portal circulation and play a key role in immune defence by phagocytosing bacteria (45). LSECs are specialised endothelial cells that line the hepatic sinusoids and form fenestrated sieve plates along the sinusoidal lumen (46). This unique structural organisation facilitates the exchange of proteins and particles between the plasma and the hepatic cells while maintaining selective barrier functions. HSC are specialized pericytes located in the space of Disse, which is a small anatomical compartment situated between hepatocytes and sinusoidal endothelial cells (47). The liver also contains smaller populations of immune cells, including B cells, T cells, NK cells, monocytes/macrophages, dendritic cells, and neutrophils (48). The liver lobule is the liver’s functional and structural unit. Each lobule is roughly hexagonal in shape and consists of a central vein at its centre, portal triads at the corners, hepatocyte plates that radiate outwards from the central vein, and sinusoids running between the hepatocyte plates towards the central vein. Based on this architecture, the liver lobule can be divided into three functional zones: the periportal (pp) zone near the portal triad; the midzone (mz) between the portal triad and the central vein; and the pericentral (pc) zone surrounding the central vein (15). A schematic representation of the liver architecture is shown in **Figure 3A**.

**Figure 4:**
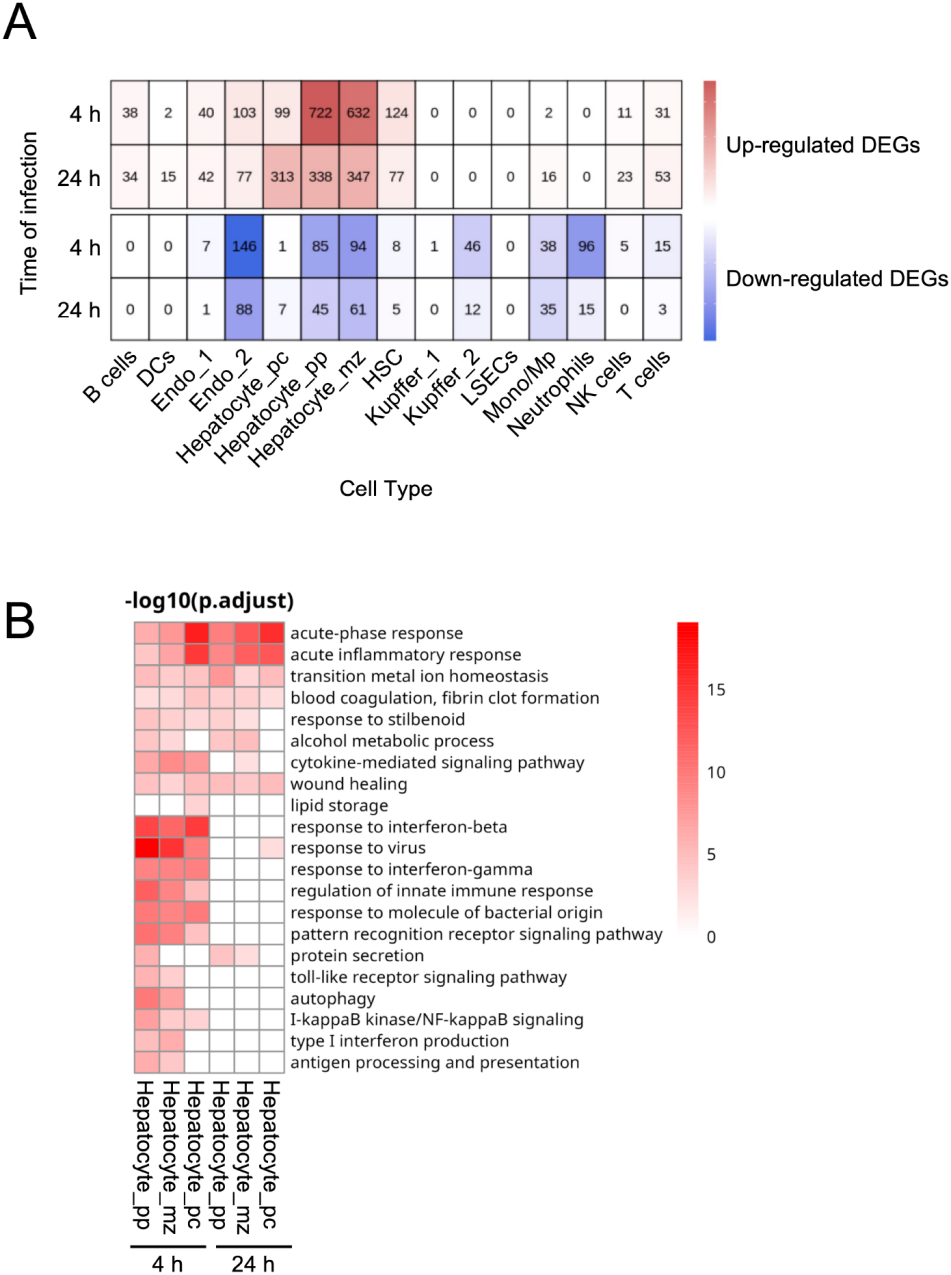
Distinct patterns of DEGs across hepatocyte populations during *S. aureus* BSI. **(A)** Heatmap showing the numbers of upregulated (red) and downregulated (blue) DEGs across liver cell populations at 4 h and 24 h after intravenous *S. aureus* infection. **(B)** Heatmap depicting selected enriched GO pathways associated with upregulated DEGs in distinct hepatocyte populations at 4 h and 24 h after bacterial inoculation, compared with uninfected controls.

To identify the liver cell populations responding to blood-born *S. aureus*, we performed single-cell RNA-sequencing (scRNA-seq) on livers collected from infected mice at 4 h and 24 h of infections, as well as from uninfected control mice. Cell cluster annotation was based on the expression of canonical marker genes (**Supplementary Figure S3** and **Figure 3B**). Analysis of transcriptomic changes associated with infection revealed that different liver cell populations responded differently to blood-borne *S. aureus* (**Supplementary Table S3** and **S4**). The UpSet plots in **Figure 3C** and **Supplementary Figure S4** summarize the size and overlap of the DEGs sets across all cell clusters at 4 h and 24 h of infection, respectively. Among the different liver cell types, hepatocytes exhibited the highest number of DEGs at both 4 h (**Figure 3C**) and 24 h (**Supplementary Figure S4**) of infection. Within the hepatocyte compartment, distinct subpopulations also responded differently to infection (**Supplementary Tables S3** and **S4**). Pericentral hepatocytes (hepatocyte_pc) displayed lower overall transcriptional activity than periportal (hepatocyte_pp) and midzone (hepatocyte_mz) hepatocytes (**Figure 3C** and **Supplementary Figure S4**). Notably, the number of upregulated DEGs in hepatocyte_pp and hepatocyte_mz was high at 4 h of infection, but declined by 24 h (**Figure 4A**). In contrast, hepatocyte_pc exhibited relatively few upregulated DEGs at 4 h, followed by a pronounced increase at 24 h (**Figure 4A**). The differences observed in the timing and magnitude of the transcriptional responses among different hepatocyte populations may reflect distinct reactions to zone-specific gradients along the porto-central axis of the liver lobule. Another striking observation was the significantly lower number of downregulated DEGs in hepatocyte_pc at both 4 h and 24 h, in contrast to the more extensive downregulation observed in hepatocyte_pp and hepatocyte_mz (**Figure 4A**).

GO enrichment analysis of DEGs that were upregulated across the different hepatocyte populations in infected mice was performed to gain deeper insight into the pathways modulated by *S. aureus* infection. As shown in **Figure 4B**, the acute-phase and acute inflammatory responses were the predominant pathways activated in all hepatocyte populations. Notably, interferon-related responses were only detected at 4 h of infection in all hepatocyte clusters, despite the high levels of IFN-γ measured in liver homogenates at 24 h (**Figure 2**). This observation is consistent with previous reports showing that the upregulation of interferon-stimulated genes occurs within 30–60 minutes of exposure to interferon and typically declines after several hours, even when interferon levels remain elevated (49–51). Thus, under sustained exposure to interferon, the interferon signaling pathway actively turns itself off through multiple negative-feedback regulatory mechanisms, as chronic, unrestrained interferon signaling can drive persistent inflammation and lead to immunopathology and tissue damage (49–51). In this regard, the genes encoding the Suppressor of Cytokine Signaling 1 (SOCS1) and 3 (SOCS3), which are potent negative regulators of both type I and type II interferon signaling pathways (51, 52), were significantly upregulated in the liver of infected mice at 24 h of infection. Specifically, SOCS1 was upregulated in endothelial cells, while SOCS3 was upregulated in hepatocyte-mz, endothelial cells, and Kupffer cells (**Supplementary Table S4**).

Immunological functions, such as toll-like receptor signaling and antigen processing and presentation, were primarily induced in hepatocyte_pp and hepatocyte_mz, but only at 4 h of infection. This zonate early response likely reflects the greater exposure of these hepatocyte subsets to blood-borne pathogens and inflammatory mediators entering through the portal circulation, as well as their proximity to resident liver immune cells such as Kupffer cells, which rapidly release cytokines upon pathogen sensing (15). This may also explain why hepatocyte_pp and hepatocyte_mz exhibited a higher number of downregulated DEGs in response to infection than hepatocytes-pc, as certain transcriptional programs need to be actively suppressed to reallocate cellular resources towards inducing of immune-protective responses. Similar spatial patterns of hepatocyte responses have been observed in the human liver, where immune response pathways are predominantly enriched in periportal regions (53).

### *Bmper* is specifically induced in periportal and midzone hepatocytes during *S. aureus* BSI

**Figure 5:**
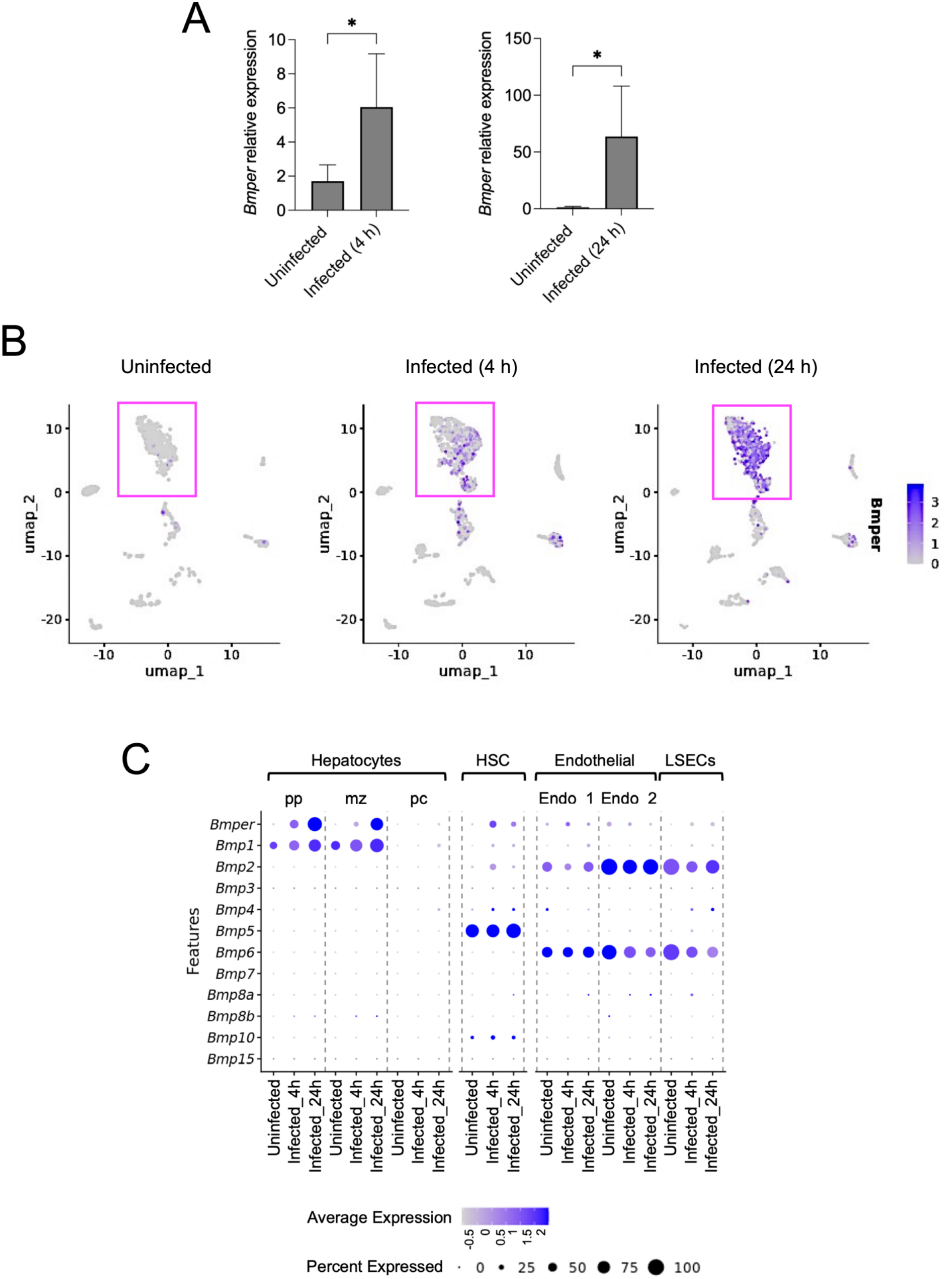
*Bmper* expression is enriched in periportal and midzone hepatocytes in the liver of *S. aureus*-infected mice. **(A)** Expression levels of *Bmper* in the liver of uninfected mice and mice infected with *S. aureus* for 4 h or 24 h, determined by RT-PCR. Data represent mean ± SD of three independent experiments. *p < 0.05. **(B)** Feature plot showing *Bmper* transcript expression across different liver cell clusters, revealing preferential expression in hepatocyte populations. **(C)** Bubble plot illustrating the expression levels of *Bmper* and genes encoding BMPs in hepatocytes, HSC, endothelial cells, and LSECs, identified in the scRNA-seq dataset. Circle size indicates the percentage of cells expressing each gene, while colour intensity reflects the average expression level.

As mentioned above, *Bmper* was among the most strongly upregulated genes identified by bulk RNA-seq in the livers of *S. aureus*-infected mice (**Figure 1D**, **Supplementary Table S1** and **S2**). The induction of *Bmper* in response to *S. aureus* BSI was confirmed by RT-PCR (**Figure 5A**). BMPER, also known as Crossveinless-2 (CV-2), is a secreted glycoprotein that functions as a key extracellular modulator of BMPs signalling (38). BMPs are multifunctional members of the transforming growth factor-beta (TGF-β) superfamily (54). Initially identified for their ability to induce bone formation (55, 56), BMPs are now recognised to regulate a broad spectrum of biological processes, including cell differentiation, proliferation, and morphogenesis across multiple tissues (54). BMPER fine-tunes the activity of BMPs in a concentration-dependent manner: at lower concentrations, BMPER enhances BMPs signaling, at higher concentrations, however, it can antagonise the functionality of BMPs by sequestering BMPs molecules and preventing their interaction with receptors (57). This dual regulatory capacity enables BMPER to locally modulate the intensity and outcome of BMPs signaling, which is crucial for controlling inflammation and vascular homeostasis. Although *Bmper* has been described as being predominantly expressed by endothelial cells (40), the scRNA-seq analysis of livers from *S. aureus-*infected mice revealed *Bmper* to be preferentially expressed by hepatocytes (**Figure 5B**), specifically by hepatocyte_pp and hepatocyte_mz, but not by hepatocyte_pc (**Figure 5C**). This zonated pattern of *Bmper* expression across the hepatic lobule is likely driven by infection-associated signaling gradients originating from the portal vein.

Regarding the expression of genes encoding BMPs, scRNA-seq analysis indicated that *Bmp2* and *Bmp6* were constitutively expressed by endothelial and LSECs cells, whereas *Bmp5* was expressed by HSC (**Figure 5C**). Interestingly, expression of *Bmp1* was upregulated in hepatocyte_pp and hepatocyte_mz during infection. Unlike other BMPs, BMP1 does not belong to the TGF-β superfamily. but is instead a metalloprotease involved in the processing of extracellular matrix components (58, 59). The proteolytic activity of BMP1 is important for developmental processes, extracellular matrix assembly, tissue remodelling, and tissue repair (60). Therefore, BMP1 produced by hepatocytes may play a key role in the immune response to *S. aureus* in the liver by remodelling the extracellular matrix and facilitating leukocyte migration into infected tissue.

### Rapid recruitment of innate immune cells into the liver during *S. aureus* BSI

**Figure 6:**
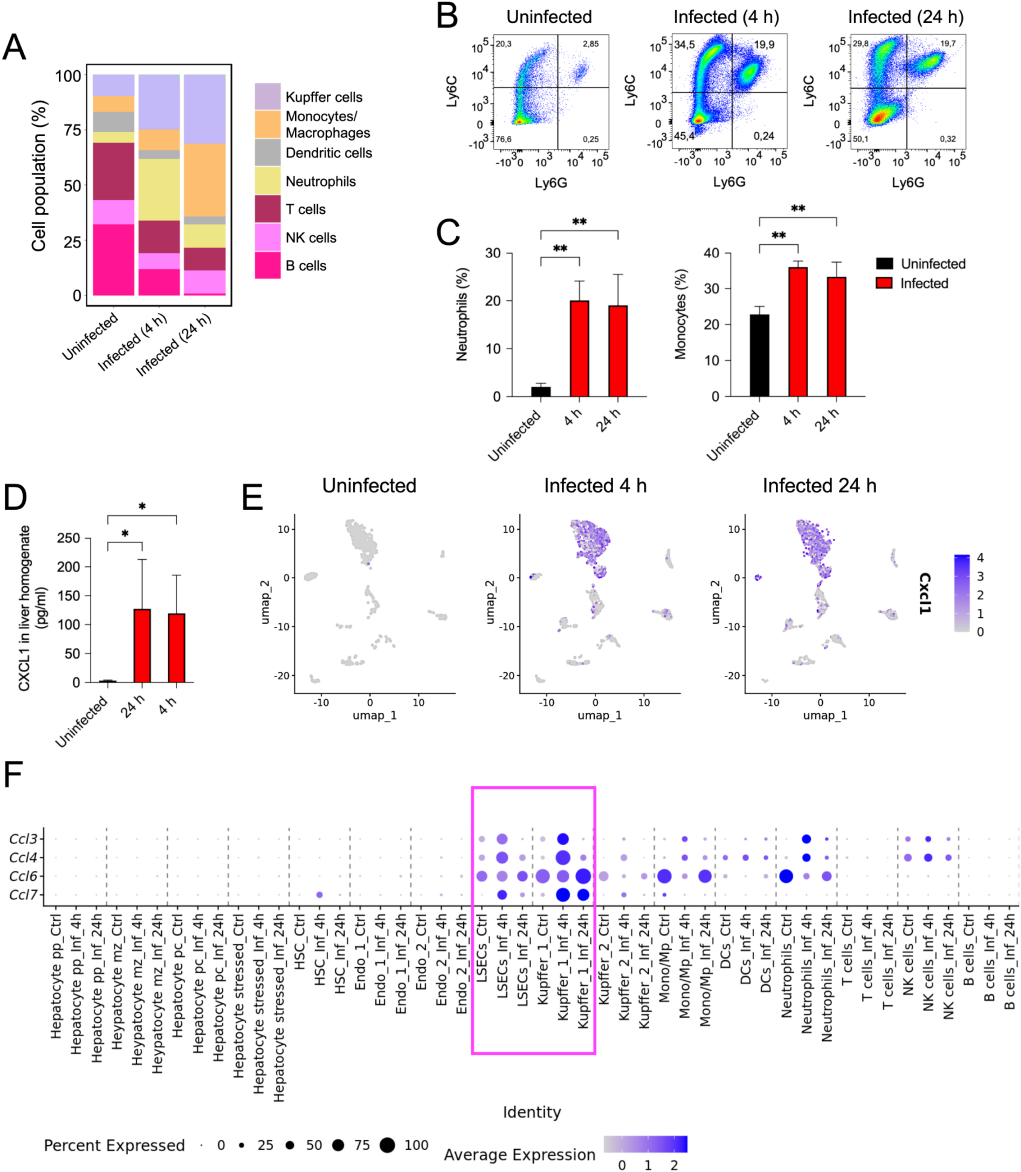
Compartmentalised expression of genes encoding chemoattractants within the liver of *S. aureus*-infected mice. **(A)** Proportions of immune cell populations in the livers of uninfected mice and mice infected with *S. aureus* for 4 h or 24 h, as determined by scRNA-seq analysis. Data are shown as a percentage of immune cells. **(B)** Flow cytometry analysis showing the frequency of monocytes/macrophages (CD11b^+^Ly6C^+^Ly6G^-^) and neutrophils (CD11b^+^Ly6C^+^Ly6G^+^) in the livers of uninfected mice and mice infected with *S. aureus* for 4 h or 24 h. The gating strategy is shown in **Supplementary Figure S1**. **(C)** Frequency of monocytes/macrophages (right) and neutrophils (left) in the livers of uninfected mice and mice infected with *S. aureus* for 4 h or 24 h. Data are presented as mean ± SD from five mice per group. Data are representative of three independent experiments. **p < 0.01. **(D)** Levels of CXCL1 in liver homogenates from uninfected or *S. aureus*-infected mice at 4 h and 24 h after bacterial inoculation. Data are presented as mean ± SD from n = 5 biological replicates. *p < 0.05. **(E)** Feature plot showing *Cxcl1* expression across cell clusters in the livers of uninfected mice and mice infected with *S. aureus* for 4 h or 24 h, revealing preferential enrichment in hepatocyte populations. **(F)** Bubble plot showing the expression levels of *Ccl3*, *Ccl4*, *Ccl6*, and *Ccl7* across different cell populations in the livers of uninfected mice and mice infected with *S. aureus* for 4 h or 24 h, as determined by scRNA-seq analysis. Circle size indicates the percentage of cells expressing each gene, and colour intensity reflects the average expression level. Cell populations expressing high levels of these genes are highlighted in a square.

The scRNA-seq analysis revealed a significant increase in the frequency of innate immune cells, including monocytes, neutrophils, and Kupffer cells, in infected livers compared to uninfected controls (**Figure 6A**). Flow cytometry analysis confirmed a marked increase in the frequency of monocytes/macrophages (CD11b^+^Ly6C^+^Ly6G^-^) and neutrophils (CD11b^+^Ly6C^+^Ly6G^+^) in the livers of infected mice (**Figures 6B, C**). These recruited innate immune cells, together with resident Kupffer cells, may be responsible for the significant reduction in bacterial counts observed in the liver between 4 and 24 h after bacterial inoculation (**Figure 1B**).

The recruitment of neutrophils and monocytes/macrophages into the infected liver is likely driven by the rapid production of chemoattractants by liver-resident cells in response to blood-borne *S. aureus.* In this regard, the level of CXCL1, a chemokine involved in the chemoattraction of neutrophils, was significantly increased in the liver in response to infection (**Figure 6D**). Interestingly, the production of chemoattractants appears to differ across liver niches. In this context, the gene encoding CXCL1, a potent neutrophil chemoattractant, was predominantly expressed by hepatocytes (**Figure 6E**), underscoring their central role in directing neutrophils to sites of infection. In contrast, genes encoding CCL chemokines, which mediate the recruitment of monocytes and macrophages, were mainly expressed by LSECs and Kupffer cells (**Figure 6F**). Together, these findings suggest the existence of a compartmentalised chemotactic signalling network within the liver during *S. aureus* BSI.

### Kupffer cell population expands in the liver during *S. aureus* BSI

**Figure 7:**
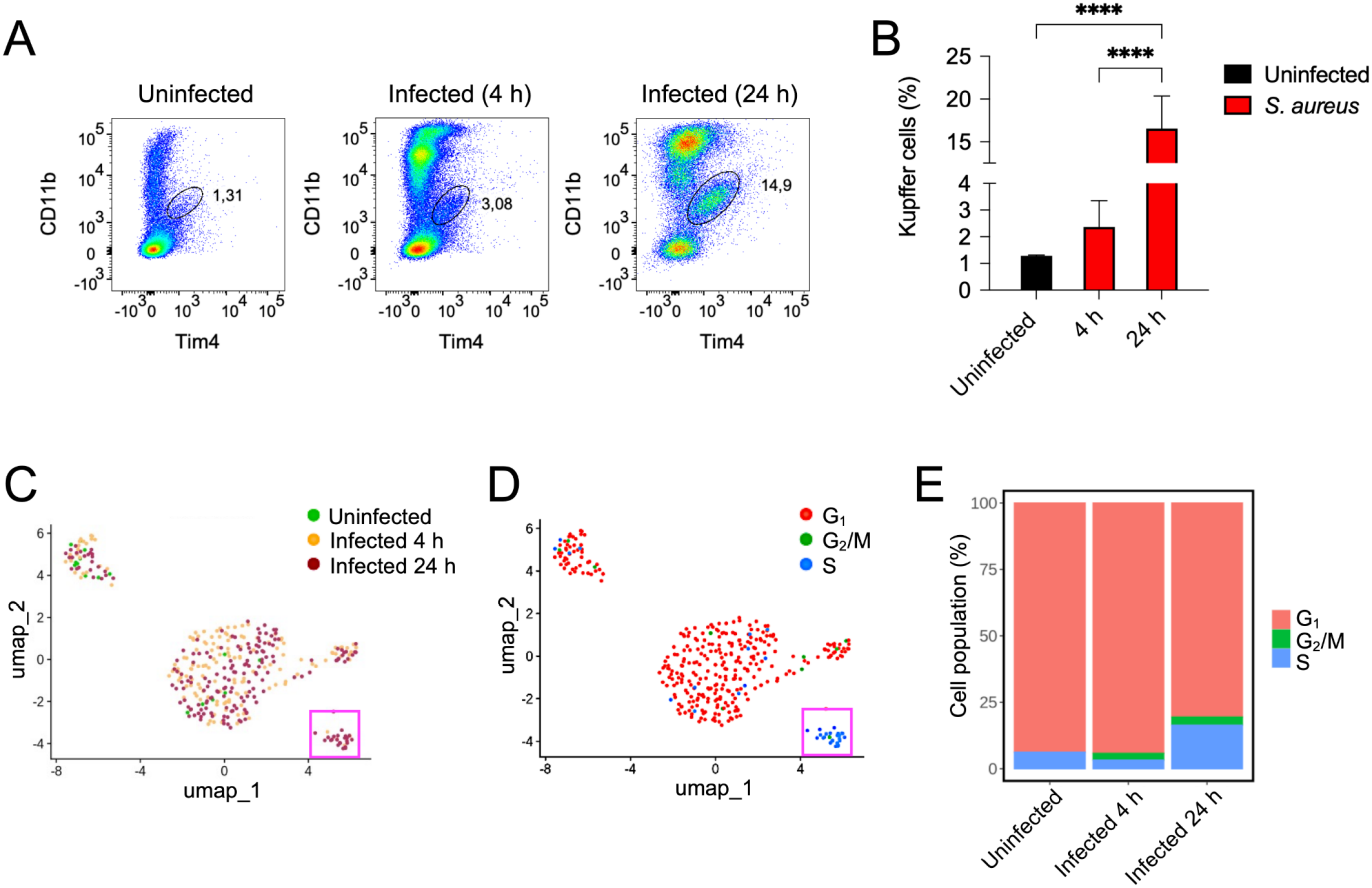
Expansion of Kupffer cells in the liver of *S. aureus*-infected mice. **(A)** Flow cytometric analysis showing the frequency of Kupffer cells (CD11b^low^Tim4^+^) in the liver of uninfected and *S. aureus*-infected mice (4 h and 24 h of infection). **(B)** Frequency of Kupffer cells in the liver of uninfected and *S. aureus*-infected mice (4 h and 24 h of infection). Data are presented as mean ± SD from five mice per group and are representative of three independent experiments. ***p < 0.001. **(C)** UMAP plot showing transcriptionally distinct Kupffer cell populations identified by scRNA-seq in liver from uninfected and *S. aureus*-infected mice at 4 h and 24 h of infection coloured by condition. **(D)** UMAP plot showing Kupffer cell populations in the different phases of the cell cycle in the liver of uninfected and *S. aureus*-infected mice (4 h and 24 h of infection). Squares highlight Kupffer cells from infected mice that are undergoing proliferation (S phase). **(E)** Proportions of Kupffer cells in the different phases of the cell cycle in the liver of uninfected and *S. aureus*-infected mice (4 h and 24 h of infection). G_1_: cell growth phase; G_2_/M: transition from the G_2_ phase (preparation for mitosis) to the M phase (mitosis); S: synthesis phase (DNA replication).

As shown in **Figure 6A**, scRNA-seq analysis revealed a significant increase in the frequency of Kupffer cells in the liver in response to infection. This finding was confirmed by flow cytometry, which demonstrated an expansion of Kupffer cells (CD11b^low^Tim4^+^) in the livers of infected mice compared to uninfected controls (**Figures 7A, B**). Kupffer cells originate from yolk sac–derived embryonic progenitors that populate the liver early in embryogenesis (61). However, during infection or inflammation, the Kupffer cell population in the adult liver can increase either through local proliferation of resident Kupffer cells or through the recruitment and differentiation of monocytes-derived macrophages (62–64). Analysis of genes associated with the G_1_, S, and G_2_/M phases of the cell cycle (65, 66) in the Kupffer cell subsets showed that, in uninfected livers, most cells were in the G_1_ phase of the cell cycle, with only very few in phase S (**Figures 7C–E**). Following infection, the proportion of Kupffer cells undergoing proliferation (S and G_2_/M phases) increased at 4 h of infection and rose even further and 24 h of infection (**Figures 7C–E**), suggesting that local proliferation of resident Kupffer may contribute, at least in part, to their increased frequency during infection.

## Discussion

In this study, we demonstrate that the liver plays a central and multifaceted role in *S. aureus* BSI, functioning both as a barrier organ for bacterial clearance and as a regulator of systemic immune responses. We further show that the liver’s response to blood-borne *S. aureus* involves a tightly coordinated interplay between different liver cell populations, compartmentalised across both spatial and cellular dimensions. Our sc-RNA-seq analysis identified hepatocyte_pp and hepatocyte_mz as the primary responders to circulating *S. aureus*, highlighting a central role for these parenchymal cells in orchestrating the early immune response during *S. aureus* BSI. Hepatocyte_pp, in particular, are anatomically positioned at the interface of the portal vein and hepatic artery, placing them in an optimal location to detect and respond to incoming pathogens (15, 16, 18). In contrast, hepatocyte_pc displayed lower overall transcriptional activity, consistent with their primarily metabolic function (15, 16, 18). This spatial asymmetry reflects a functional specialization that may have evolved to balance immune surveillance with metabolic homeostasis. Supporting this concept, it has been reported that human hepatocytes from different lobular zones differ in their permissiveness to *Plasmodium* liver-stage infection, demonstrating that lobular spatial positioning can modulate hepatocyte susceptibility to blood-borne pathogens (67).

Hepatocytes are the main cells responsible for synthesising and secreting acute-phase proteins such as serum amyloids (SAA), C-reactive protein (CRP), complement components, haptoglobin, transferrin, and hepcidin, among others (10–12). These proteins play essential roles in enhancing the immune response and promoting pathogen clearance (10–12). The synthesis of acute-phase proteins by hepatocytes is generally induced by inflammatory cytokines such as IL-6, TNF-α, and IL-1β (42, 68). In our study, we detected significant levels of these cytokines in the liver as early as 4 h of infection. The likely source of these cytokines was activated Kupffer cells in response to *S. aureus*. Previous studies have indeed shown that Kupffer cells can produce several proinflammatory cytokines in response to bacterial products (62, 63, 69).

One of the key findings of our study was that *Bmper* was among the genes that were most strongly upregulated in the liver during *S. aureus* BSI. Although BMPER has traditionally been associated with endothelial cell biology and vascular development (38–40, 70), it has not previously been implicated in bacterial infections. BMPER functions as a regulator of BMPs, and multiple studies have reported that the BMPs signalling pathways are regulated during endothelial dysfunction and inflammation (71–73). Notably, BMPER is essential for development, as homozygous knockout mice (Bmper^−/−^) are embryonically lethal (74). Endothelial leakage is one of the earliest events in acute inflammation, allowing plasma proteins, fluid, and immune cells to move into infected tissue (75). Several studies have demonstrated a protective role of BMPER, showing that it exerts anti-inflammatory effects, inhibits vascular inflammation, and preserves endothelial integrity (39, 70, 76–79). Based on these facts, we hypothesise that BMPER may contribute to maintaining endothelial integrity during *S. aureus* infection by regulating the activity of BMPs, thereby limiting excessive vascular permeability and widespread inflammation. Furthermore, the capacity of BMPER to modulate inflammatory signalling suggests that it may influence the recruitment and activation of immune cells to the infected tissue. Indeed, BMPER has been shown to modulate leukocyte adhesion and migration in both *in vitro* and *in vivo* studies (39). Further research is needed to fully elucidate how BMPER modulates immune and vascular responses during *S. aureus* BSI.

In our study, we observed a significant reduction in bacterial load in the liver between 4 h and 24 h of infection. This reduction is likely attributable to the activity of resident Kupffer cells, as well as recruited phagocytic cells such as monocytes and neutrophils, as previously reported (26, 27). Consistent with this, flow cytometry analysis of liver cells demonstrated an increase in monocytes/macrophages and neutrophils as early as 4 h of infection. The recruitment of phagocytic cells is generally mediated by chemoattractants. In this context, the gene encoding the chemokine CXCL1, which is involved in neutrophil recruitment, was found to be upregulated in hepatocytes. In contrast, genes encoding CCL chemokines, which are typically involved in the recruitment of monocytes and macrophages, were found to be upregulated in LSECs and Kupffer cells. This distinct cellular distribution of chemoattractant gene expression indicates a coordinated spatial organisation of chemokine production within the infected liver. This organization may facilitate the targeted recruitment of specific immune cell subsets to defined hepatic compartments during infection. The production of CXCL1 by hepatocytes may be induced by TNF-α derived from Kupffer cells, as it has been shown that TNF-α from Kupffer cells is essential for the expression of CXCL1 by hepatocytes and subsequent recruitment of neutrophils following a challenge with necrotic cells (80).

One particularly interesting observation in this study was the expansion of Kupffer cells in the livers of infected mice. Two major mechanisms can contribute to an increase in Kupffer cell numbers during infection or tissue damage: local proliferation of resident Kupffer cells and/or differentiation of recruited circulating monocytes into Kupffer cells (62–64). Several models of liver injury that result in Kupffer cell depletion have shown that bone marrow-derived monocytes infiltrate the liver and adopt the Kupffer cell phenotype in order to repopulate the vacant niche (81–83). Conversely, local proliferation of endogenous Kupffer cells has been demonstrated in models of acute injury and partial hepatectomy, a process that appears to be driven by IL-6 signalling (83, 84). In our model of *S. aureus* BSI, we found that a proportion of Kupffer cells in infected mice expressed genes associated with the G_2_/M and S phases of the cell cycle, indicating that the increased frequency of Kupffer cells in the livers of infected mice may result, at least in part, from the local proliferation of resident Kupffer cells. This is most likely as a compensatory response to the loss of Kupffer cells caused by *S. aureus*-induced cell lysis (27, 85).

In summary, our findings reveal previously unrecognized aspects of the early immune responses activated within the liver during *S. aureus* BSI. We demonstrate that the hepatic immune reaction, including the transcriptional activation of acute-phase proteins, the initiation of the phagocytic cell recruitment, and the regulation of BMPs-modulating pathways, is highly compartmentalised. Specifically, periportal and midzonal hepatocytes orchestrate the initial immune response against circulating *S. aureus*. Such as spatial coordinated activity likely promotes efficient bacterial clearance while maintaining controlled inflammation, thereby limiting collateral tissue damage. Moreover, the identification of *Bmper* as a zonally induced regulator establishes a mechanistic link between vascular signalling and hepatic immunity. Further investigation into the role of BMPER and related pathways may uncover new opportunities to enhance liver-specific immunity or mitigate inflammation-driven liver injury during BSI.

## Data Availability

The datasets presented in this study can be found in online repositories. The names of the repository/repositories and accession number(s) can be found in the article.
