# Supplementary material for: Zone-specific hepatocytes orchestrate the early onset of host immune defenses during Staphylococcus aureus bloodstream infection
Source: Front Immunol. 2026 Apr 30;17:1776887. doi: 10.3389/fimmu.2026.1776887 (PMC13171408; doi:10.3389/fimmu.2026.1776887)
Supplement: Supplementary file 1 [file DataSheet1.pdf]

Supplementary Figure S1

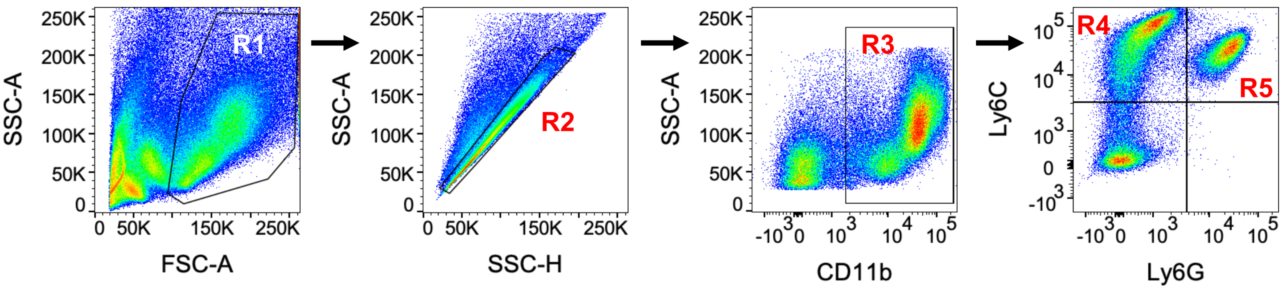

Figure S1. Flow cytometry gating strategy for identification monocytes/macrophages (CD11b$^+$Ly6C$^+$Ly6G$^-$) and neutrophils (CD11b$^+$Ly6C$^+$Ly6G$^+$) in the liver. Representative plots show sequential gating steps from total cells to the final Ly6C$^+$Ly6G$^+$ subset. Single-cell suspensions were first gated based on forward scatter to exclude debris (R1). This is followed by subsequent gates using SSA-A vs. SSC-H to exclude doublets (R2). CD11b$^+$ events were selected (R3) and, within this population, Ly6C and Ly6G expression were analyzed to distinguish monocytes/macrophages (Ly6C$^+$Ly6G$^-$) from neutrophils (Ly6C$^+$Ly6G$^+$).

Supplementary Figure S2

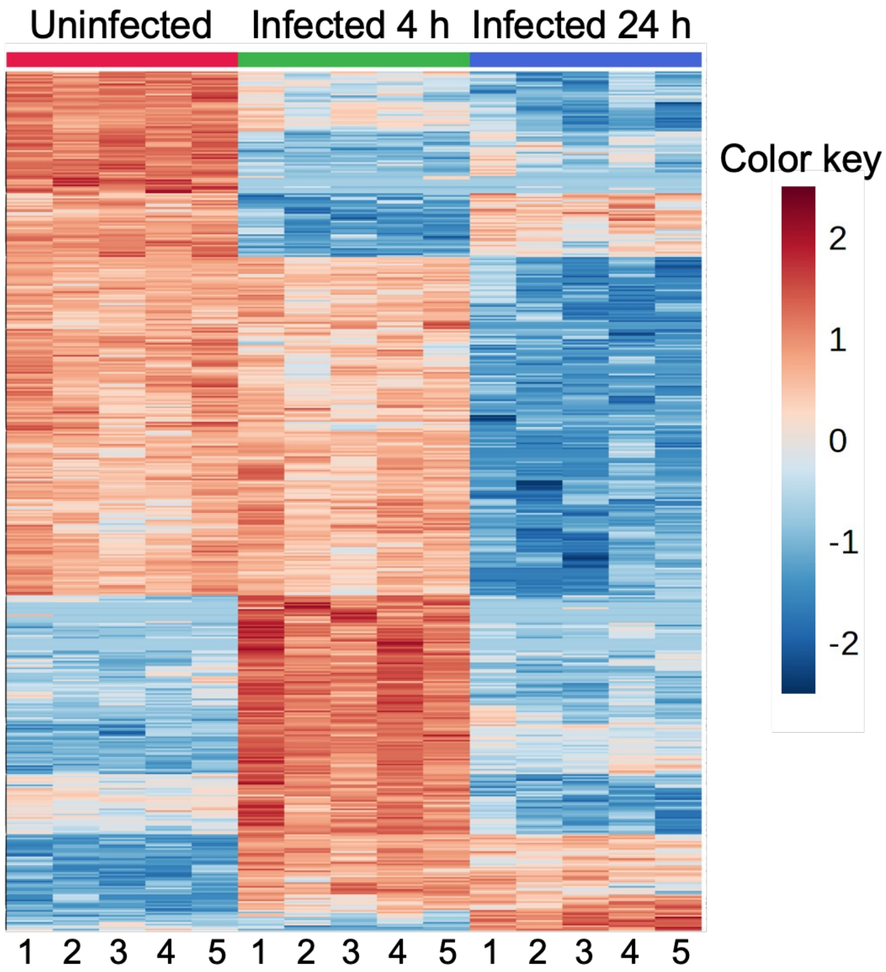

Figure S2. Heatmap of liver gene expression levels following *S. aureus* bloodstream infection. Heatmap depicting differential gene expression levels in liver samples from uninfected mice and mice intravenously infected with *S. aureus* at 4 and 24 h post-inoculation. Each column represents an individual biological replicate, and each row corresponds to a gene. Gene expression values were normalized and scaled by Z-score. Expression levels are normalized and color-coded according to relative transcript abundance (high, red; low, blue).

Supplementary Figure S3

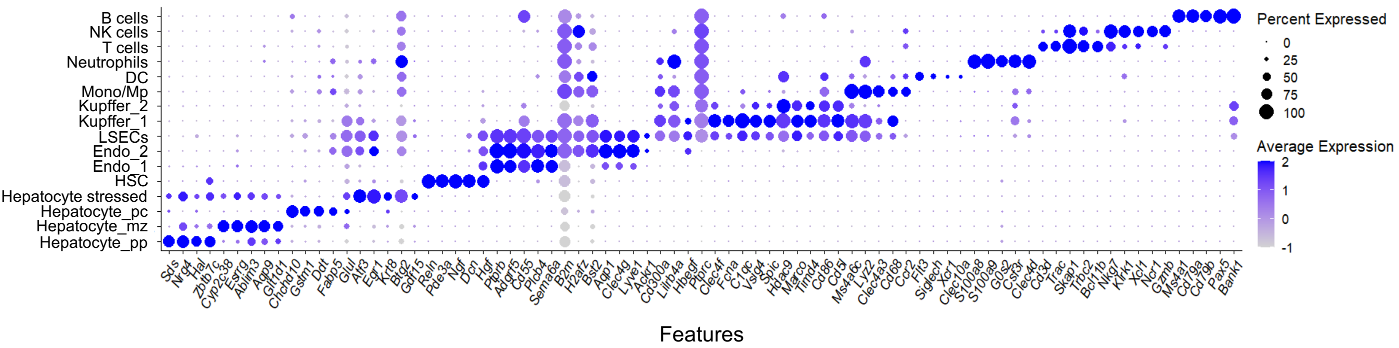

Figure S3. Annotation of cell clusters from scRNA-seq analysis. Cell clusters were annotated based on the expression patterns of canonical marker genes characteristic of specific cell types.

Supplementary Figure S4

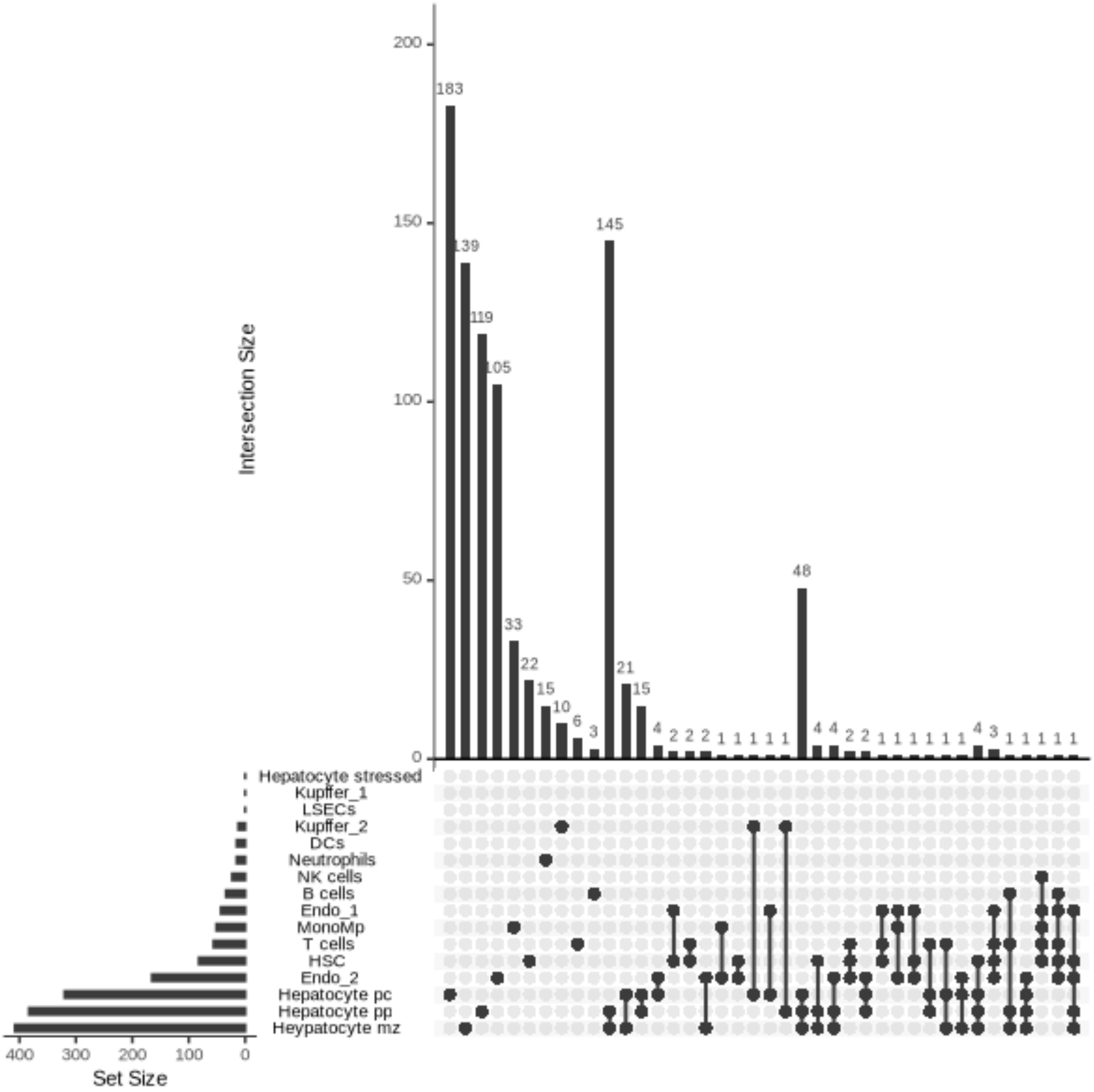

Figure S4. UpSet plot of DEGs across the different liver cell populations in response to blood-borne *S. aureus* infection at 24 h after intravenous inoculation. The lefts bar chart indicates the total number of DEGs per cell population, while the dot-matrix shows overlapping DEG combinations between cell populations. Rows with filled dots under two or more columns denote genes shared among multiple populations and connected filled dots indicate common genes among the highlighted populations. The top bar chart represents the size of each intersection.
